# Obesity Heterogeneity by Neighborhood Context in a Largely Latinx Sample

**DOI:** 10.1007/s40615-023-01578-6

**Published:** 2023-03-30

**Authors:** Ashley W. Kranjac, Dinko Kranjac, Zeev N. Kain, Louis Ehwerhemuepha, Brooke N. Jenkins

**Affiliations:** 1 Department of Sociology, Chapman University, Orange, CA USA; 2 Center for Stress & Health, University of California School of Medicine, Irvine, CA USA; 3 Department of Psychology, Institute for Mental Health and Psychological Well-Being, University of La Verne, La Verne, CA USA; 4 Department of Anesthesiology and Perioperative Care, University of California, Irvine, CA USA; 5 Yale Child Study Center, Yale University, New Haven, CT USA; 6 Computational Research, Children’s Health of Orange County, Orange, CA USA; 7 Department of Psychology, Chapman University, One University Drive, Orange, CA 92866 USA

**Keywords:** Children, Electronic medical records, Latent profile analysis, Latinx, Neighborhoods, Obesity

## Abstract

Neighborhood socioeconomic context where Latinx children live may influence body weight status. Los Angeles County and Orange County of Southern California both are on the list of the top ten counties with the largest Latinx population in the USA. This heterogeneity allowed us to estimate differential impacts of neighborhood environment on children’s body mass index *z*-scores by race/ethnicity using novel methods and a rich data source. We geocoded pediatric electronic medical record data from a predominantly Latinx sample and characterized neighborhoods into unique residential contexts using latent profile modeling techniques. We estimated multilevel linear regression models that adjust for comorbid conditions and found that a child’s place of residence independently associates with higher body mass index *z*-scores. Interactions further reveal that Latinx children living in Middle-Class neighborhoods have higher BMI *z*-scores than Asian and Other Race children residing in the most disadvantaged communities. Our findings underscore the complex relationship between community racial/ethnic composition and neighborhood socioeconomic context on body weight status during childhood.

## Introduction

Childhood obesity is a major public health challenge in the USA [1]. The prevalence of obesity among children aged 2–19 varies by race/ethnicity, and the number of Latinx children and adolescents who are presenting with obesity is increasing [2–4]. Latinx youth represent one-quarter of the entire population of children under the age of 18 in the USA and are disproportionally burdened by obesity [5, 6]. A continuing rise in the prevalence of obesity among Latinx children is a serious concern because an excess of body fat is associated with adverse effects on health that may persist into adulthood such as hypertension, prediabetes and diabetes mellitus, sleep apnea, asthma, and depression [1, 6–8]. Although the prevalence varies among Latinx Americans of different ethnic backgrounds regardless of origin group, Latinx adults are more likely to be diagnosed with these medical conditions, as compared to their non-Latinx White counterparts [9–13].

Unhealthy weight gain develops from a chronic, positive energy balance through an interplay of genetic, biological, behavioral, socioeconomic, and environmental factors [14–16]. For example, underlying medical causes such as poisoning, infections, respiratory or heart disease, and immune system, central nervous system, or endocrine system dysfunction may lead to obesity [17–23]. Also, children from low socioeconomic status (SES) families are more likely to be diagnosed with obesity, and, predictably, this is true among Latinx youth in general and in California, specifically [24, 25]. Relatedly, the consumption of high-calorie foods with little-to-no nutritional value and the chronic psychosocial stress brought on by having limited economic resources influence weight trajectories among Latinx children [26–28]. Moreover, the neighborhood socioeconomic environment where Latinx children live influences body weight status, and neighborhoods with predominately racial/ethnic minority residents have higher levels of poverty and lower levels of education, which are independently associated with childhood obesity [29–35]. Notably, data indicate that living in either a low-SES household or a low-SES neighborhood is enough to increase the risk of child obesity [36]. Multiple domains of the social neighborhood environment also are relevant to the development of overweight and obesity [37]. For example, social cohesion and collective socialization and trust in Latinx communities are some of the more pertinent factors because they can impact norms regarding diet and physical activity [38]. Regarding the home and family environment, the parents of Latinx youth tend to underestimate their child’s weight status and perceive their child as “normal” weight even when the child is classified as overweight or obese according to body mass index (BMI) categories [39]. Taken together, these multiple influences enhance the risk for obesity among children in general, as well as in Latinx children specifically, a population where prevalence is increasing especially rapidly [2–6].

Obesity is a multifactorial disease, and thus, we need to appreciate fully the relevance of how socioenvironmental factors like residential context combine to associate with increased body weight. As but a single example, findings from the *Moving to Opportunities Study* conducted by the Department of Housing and Urban Development indicate that relocation to a low-poverty census tract led to lower obesity prevalence [40]. One way to address the significance of multifactorial neighborhood-level determinants of obesity is to use novel methods on unique populations to draw out the extent to which distinctive residential contexts give rise to social patterning that produces variation in obesity prevalence [31, 32]. Indeed, our data source allows us to overcome some of the methodological challenges plaguing the current literature, including an objective collection of height/weight measures, access to medical diagnoses, adequate sample size and number of children per residential tract, and racial/ethnic diversity [41]. Specifically, we geocoded pediatric medical record data for residents in Southern California, a geographical area with the largest Latinx population in the USA [42]. Moreover, as a geographic setting, Los Angeles County has the nation’s largest Latinx population, while Orange County ranks tenth among the counties with the largest Latinx population in the USA [42]. We took advantage of this heterogeneity to estimate the differential impacts of neighborhood context on children’s BMI *z*-scores. First, we explored the relationship between neighborhood context and BMI *z*-scores. Given past work demonstrating an association between neighborhood socioeconomic status and children’s body weight, we expected that neighborhood disadvantage would associate with heightened BMI *z*-scores. In addition, we expected race/ethnicity would moderate the association between neighborhood context and BMI *z*-scores, and that Latinx children will have higher body weight relative to other race/ethnicities irrespective of contextual factors.

## Methods

### Data Sources

We use a compilation of data from multiple sources. We extracted electronic medical records (EMR) data from children (*n* = 53,735) between the ages of 0 and 18 admitted between June 2013 and June 2018 as inpatients across a variety of care settings. EMR data were excluded for pediatric cancer patients (*n* = 1319), those without complete body mass index (BMI) measures (*n* = 3921), and outlier patients for the length of stay (*n* = 529) [40]. Patient addresses from the remaining EMR data were geocoded and linked to the corresponding residential Census tract. To capture the appropriate time period for analysis, neighborhood measures were assigned to children temporally by first taking the child’s address from the electronic medical record at the time of their height and weight measurement. Then, we used 5-year ACS estimates for the census tracts which “surround” the timing of the child’s records. In this way, the 5-year ACS estimates characterize the child’s area within that 5-year period. As previously done, we used Census tracts to represent neighborhoods [43]. Social and economic indicators were extracted from the 2014–2018 American Community Survey (ACS) data. We excluded observations unmatched to Federal Information Processing Series (FIPS) Codes (*n* = 7610), those living in Census tracts with fewer than 20 children per tract (*n* = 32,234), and children living outside of California (*n* = 90), resulting in a total sample size of 8092 children nested within 672 census tracts or neighborhoods.

### Variables

The key outcome is age- and sex-specific BMI *z*-score (i.e., the number of standard deviation (SD) units that the child’s BMI deviates from the age- and sex-normed mean reference value, based on the 2000 Center for Disease Control (CDC) Growth Charts: USA) [44, 45]. We included all covariates available to us from the EMR to represent the child and familial characteristics. Sociodemographic characteristics include age at the time of visit, sex (1, girl; ref.; 2, boy), race/ethnicity (1, Latinx, ref.; 2, non-Latinx White; 3, non-Latinx Black; 4, Asian; 5, Other Race), and insurance type (1, Private, ref.; 2, Public (e.g., MediCare or MediCal)) as a proxy for socioeconomic status (SES) [31, 46–48]. Other measures include admission type (1, elective, ref.; 2, emergency; 3, other (i.e., urgent care or accident)); and diagnosis based on ICD9/10 codes (0, absence of diagnosis; 1, presence of diagnosis), including bacterial and viral infection (A00-A99), diseases of the blood and blood-forming organs, and disorders involving immune mechanisms (D50-D89), endocrine, nutritional and metabolic diseases (E00-E89), ref., diseases of the circulatory system (I00-I99), nervous system diseases (G00-G99), congenital malformations, deformations and chromosomal abnormalities (Q00-Q99), diseases of the digestive system (K00-K95), diseases of the genitourinary system (N00-N99), diseases of the musculoskeletal system (M00-M99), diseases of the respiratory system (J00-J99), injury, poisoning, and certain other external consequences (S00-T88), and other diagnoses (H00-H59, L00-L99, O00-O9, P00-P96, Z00-Z99). We also included length of stay (mean = 3.82; Std. Dev. = 5.95) and patient maximum pain assessed by healthcare providers throughout the child’s hospital stay using several developmentally and situationally appropriate measurement tools, including faces, legs, activity, cry, and consolability scale, faces pain scale, numeric rating scale, and neonatal pain, agitation and sedation scale (range from 0, no pain to 10, severe pain). Social and economic indicators of the child’s neighborhood of residence come from the ACS and include community-level education (% adults with < 12 years of education; % adults with 12 years of education; % adults with > 12 years and < 16 years of education; % adults with 16 years of education; % adults with 18 years of education; % adults with > 18 years and < 21 years of education), median community-level income, percent of female-headed households, percent receiving public assistance, percent in poverty, percent of homes in the tract that are rented, and racial/ethnic composition measured by percent of major racialized categories (% non-Latino White; % non-Latino Black; % Latinx; % Asian; % Other Race). After testing the cross-level assumption that the random effect of the intercept is correlated with a level 1 measure, using a Hausman test, we uncovered that some unobservable neighborhood characteristics relegated to the error term are correlated with some observable patient characteristics. We tested for correlations between neighborhoods and patient-level characteristics and found that the offending explanatory measures were race/ethnicity and insurance type. Then, we computed neighborhood aggregates of these patient-level characteristics (i.e., means of race/ethnicity and insurance type) to control for neighbor effects and included these measures at level 2 in our fully specified models [49].

### Statistical Analysis

**Fig. 1:**
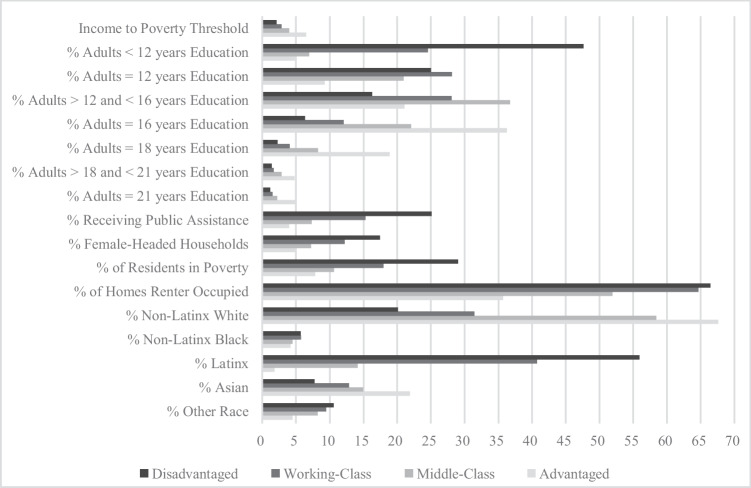
Descriptive neighborhood-level characteristics by LPA-generated neighborhood profiles

We first used latent profile analysis (LPA) to group neighborhoods into clusters based on the above-listed socioeconomic indicators extracted from the ACS, which are often used to define a child’s neighborhood of residence [50, 51]. Using MPlus 8.8 software, we estimated a 1-profile model in a type I covariance structure and fit successive models with an increasing number of profiles to characterize neighborhoods [52, 53]. We used entropy and theoretically driven evidence to select the most appropriate number of profiles and further evaluated model fit with AIC, BIC, a-BIC, and loglikelihood values to identify the most parsimonious model. Specifically, our substantive neighborhood cluster interpretations are based on theoretical neighborhood stratification observed across the USA (i.e., upper class, middle class, working class, and disadvantaged communities). The stability of our 4-profile solution was verified by model fit statistics wherein we stopped adding profiles once the model fit indicators began to increase and the entropy levels began to decrease (Table 1). Given our data, analyses indicated that neighborhoods are most appropriately captured by a 4-profile solution (Table 1). Figure 1 displays the patterning of neighborhood-level characteristics by LPA-generated neighborhood profiles. We standardized all measures for this figure by converting median household income to the ratio of income to poverty using 2016-inflation adjusted dollars for a 3-person household.

**Table 1:** Model fit & usefulness information for LPAs with 1−5 latent profiles

Classes	AIC	BIC	a-BIC	LL	LL *p*-value	Entropy
1	2949364.575	2949654.352	2949539.946	–1474646.29	0.000	1.00
2	2866031.981	2866474.696	2866299.908	–1432960.99	0.000	0.93
3	2816267.306	2816862.958	2816627.789	–1408059.65	0.000	0.92
**4**	**2781335.073**	**2782083.663**	**2781788.113**	–**1390574.54**	**0.000**	**0.95**
5	2815388.178	2816289.707	2815933.774	–1407582.09	0.333	0.91

Source: Data are from electronic medical records and the 2014−2018 American Community Survey. *Note:* Bolded row indicates the most parsimonious model

We then test the influence of distinct neighborhoods on children’s BMI *z*-scores using multilevel linear regression modeling with Stata 16.1 software [54]. This technique treats level-1 children as nested within level-2 neighborhoods and neutralizes the lack of independence of data within higher groups. Our modeling approach uses adaptive quadrature to adjust for problems that otherwise downwardly bias estimated standard errors, including different sample sizes for level-1 and level-2 units, clustering within neighborhoods, variable numbers of cases within level-2 units, and heteroscedastic error terms [55]. We performed a series of conditional models that first included the covariates of a child- and family-level predictors (age, sex, race/ethnicity, insurance type, admission type, diagnosis, length of stay, patient-reported pain levels) to test the influence of child and family factors on children’s BMI *z*-scores. In the next set of models, we included the LPA-constructed neighborhoods and neighborhood averages of race/ethnicity and insurance status at the neighborhood level (and a neighborhood-level error component) along with the child- and family-level predictors and an individual error term. In our last set of models, we included interactions between the LPA-constructed neighborhoods and race/ethnicity at the child/family level to determine whether race/ethnicity moderates the association between neighborhood disadvantage and the expected heightened BMI *z*-scores for Latinx children.

### Results

**Fig. 2:**
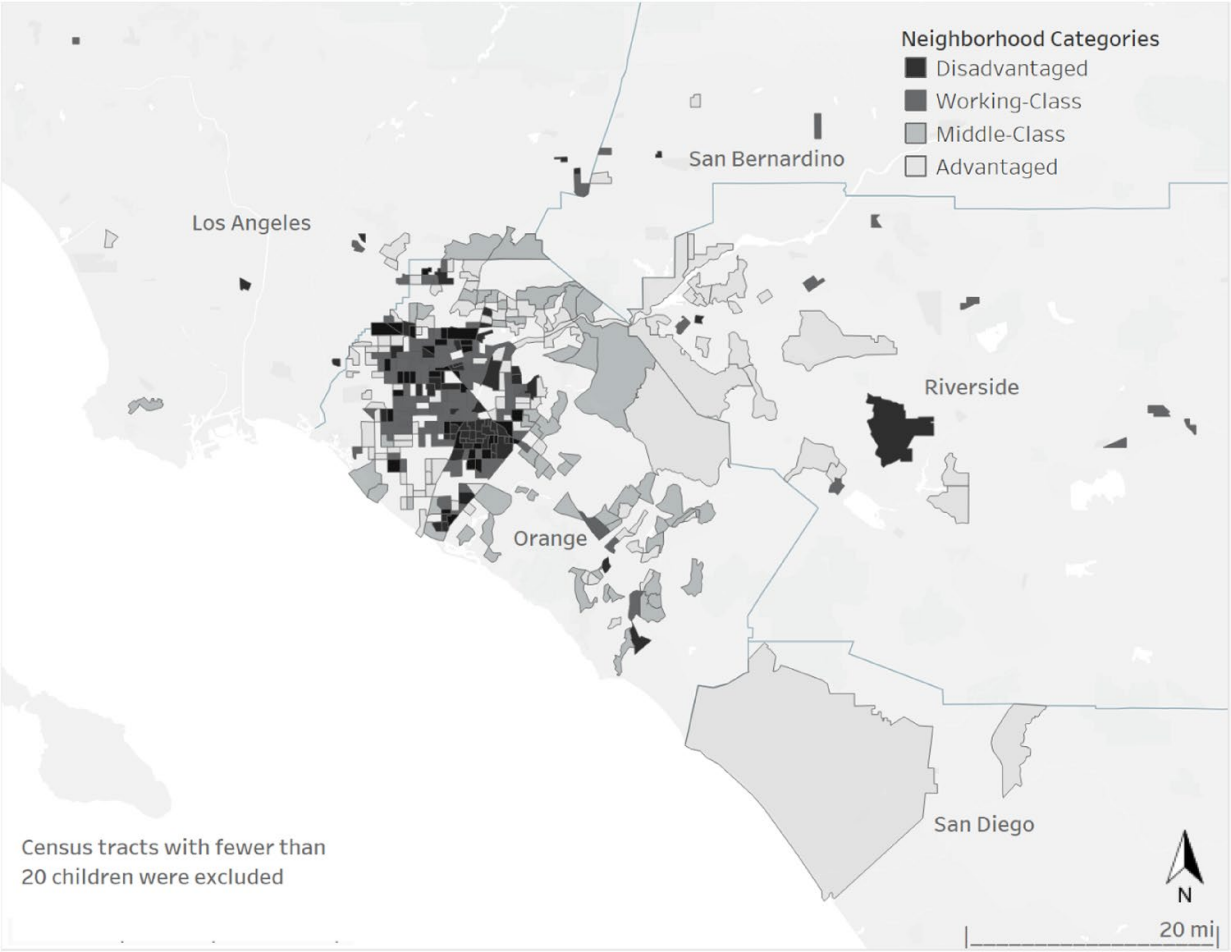
Neighborhood profiles by census tracts, Southern California

We display how the four neighborhood profiles cluster across the area of study in Fig. 2. We assigned the following labels: Advantaged, Middle-Class, Working-Class, and Disadvantaged based on the descriptive characteristics. The advantaged neighborhood profile is largely concentrated in east Orange County, north-west Riverside County, and north-west San Diego County regions. As shown in Table 2, Advantaged neighborhoods have the highest median household income ($130,461), the highest overall levels of education (31% of residents have at least 16 years of education), and the lowest percentage of people living in poverty (8%). The Disadvantaged communities make up the areas around north-west Orange County and central Riverside County regions and score the worst on nearly every indicator. These communities have the lowest median household income ($41,470), the lowest education levels (48% of adult residents lack a high school degree), and the highest proportion of the population in poverty (29%).

**Table 2:** Descriptive neighborhood-level characteristics by LPA-generated neighborhood profiles

	Advantaged		Middle-Class		Working-Class		Disadvantaged		Diff
		95% CI		95% CI		95% CI		95% CI	
Socioeconomic proportions									
Median income (in $10 K)	130.46	(128.64–133.32)	79.83	(78.86–81.36)	56.78	(56.14–57.78)	41.47	(40.67–42.72)	< .001
% adults < 12 years of education	4.83	(4.70–5.04)	6.96	(6.41–6.97)	24.54	(24.32–25.95)	47.63	(47.28–48.96)	< .001
% adults = 12 years of education	9.23	(9.19–9.67)	20.96	(20.62–21.44)	28.10	(27.89–28.48)	25.00	(24.69–25.49)	< .001
% adults > 12 and < 16 years of education	21.05	(21.02–21.86)	36.73	(36.27–36.94)	28.04	(29.05–31.05)	16.30	(16.18–20.04)	< .001
% adults = 16 years of education	36.25	(36.03–36.60)	22.06	(21.66–22.67)	12.05	(11.78–12.48)	6.30	(6.26–6.72)	< .001
% adults = 18 years of education	18.86	(18.58–19.31)	8.25	(8.08–8.52)	4.07	(3.95–4.24)	2.23	(2.15–2.34)	< .001
% adults > 18 and < 21 years of education	4.74	(4.59–4.97)	2.83	(2.77–2.92)	1.70	(1.65–1.78)	1.37	(1.29–1.49)	< .001
% adults = 21 years of education	5.04	(4.92–5.23)	2.21	(2.16–2.29)	1.50	(1.44–1.60)	1.17	(1.16–1.42)	< .001
% receiving public assistance	3.98	(3.87–4.16)	7.31	(7.11–7.62)	15.29	(14.96–15.82)	25.09	(24.54–26.21)	< .001
% female-headed households	5.02	(4.92–5.19)	7.19	(7.05–7.42)	12.19	(11.94–12.57)	17.43	(17.01–18.10)	< .001
% of residents in poverty	7.84	(7.61–8.20)	10.60	(10.35–10.99)	17.96	(17.59–18.53)	29.03	(28.30–30.18)	< .001
% of homes renter occupied	35.68	(34.81–37.06)	64.66	(35.72–37.67)	51.93	(51.13–53.17)	66.46	(65.43–68.09)	< .001
Racial/ethnic composition									
% Non-Latinx White	67.58	(67.00–68.50)	58.39	(57.81–59.29)	31.41	(30.76–34.43)	20.11	(20.38–23.26)	< .001
% Non-Latinx Black	4.17	(4.54–5.09)	4.48	(4.17–4.95)	5.72	(5.32–5.35)	5.69	(5.26–5.37)	< .01
% Latinx	1.79	(1.52–1.22)	14.14	(13.67–14.89)	40.73	(40.43–46.77)	55.93	(50.67–56.37)	< .001
% Asian	21.89	(21.37–22.72)	14.96	(14.52–15.64)	12.82	(12.35–13.56)	7.73	(6.89–7.93)	< .001
% Other Race	4.47	(4.32–4.70)	8.22	(8.02–8.55)	9.45	(9.72–9.60)	10.58	(10.89–15.65)	< .001
Neighborhoods *n* =	94		198		218		162		
Children *n* =	1159		1749		2265		2838		

Source: Data are from the 2013–2018 electronic medical records and the 2014–2018 American Community Survey (ACS)

Significance is evaluated using one-way MANOVA with the neighborhood variables as the dependent variables and LPA neighborhood type as the independent variable

**Fig. 3:**
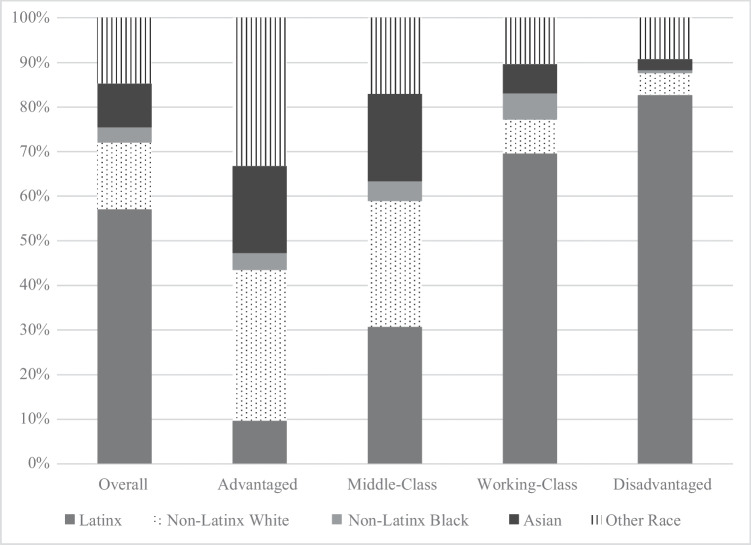
Distribution of children across LPA neighborhood contexts by race/ethnicity

We show descriptive information for the child and family characteristics overall, and by neighborhood context in Table 3. The average overall BMI is 0.38 SDs above the national reference. However, the majority of children fall at or below the 67th percentile, or in the “Healthy Weight” category. Children living in Advantaged neighborhoods have 0.02 SDs below the mean while those residing in Disadvantaged neighborhoods have 0.65 SDs above the mean. The mean age for the entire sample was 9.80 years, with children in Advantaged neighborhoods slightly older than those in other neighborhood types. In Fig. 3, we show that, although there is some clustering of children of specific race/ethnicity across neighborhoods, each racial/ethnic group is represented within each neighborhood. As displayed in Table 3, children admitted to the emergency department and presenting with diseases of the digestive system are slightly over-represented in Disadvantaged neighborhoods compared to those residing in other neighborhoods.

**Table 3:** Means and standard deviations (SD) for independent and dependent variables overall and by neighborhood profile

	Overall		Advantaged		Middle-Class		Working-Class		Disadvantaged		Sig
	Mean or %	SD	Mean or %	SD	Mean or %	SD	Mean or %	SD	Mean or %	SD	
Dependent variable											
BMI *z*-scores	0.38	1.49	− 0.02	1.31	0.17	1.65	0.41	1.49	0.65	1.41	< .01
Independent variables											
Sociodemographic											
Age	9.80	4.90	10.34	4.83	9.44	5.01	9.77	4.82	9.86	4.91	< .01
Sex											
Girl	48.00	0.50	40.38	0.49	49.80	0.50	48.96	0.50	50.00	0.50	
Boy	52.00	0.50	59.62	0.49	50.20	0.50	51.04	0.50	50.00	0.50	
Race/ethnicity											
Latinx	56.90	0.50	9.58	0.29	30.65	0.46	69.58	0.46	82.70	0.38	< .001
Non-Latinx White	15.24	0.36	33.91	0.47	28.30	0.45	7.68	0.27	4.97	0.22	< .001
Non-Latinx Black	3.42	0.18	3.80	0.19	4.46	0.21	5.87	0.24	0.70	0.08	
Asian	9.76	0.30	19.50	0.40	19.56	0.40	6.53	0.25	2.40	0.15	< .001
Other Race	14.68	0.35	33.22	0.47	17.04	0.38	10.33	0.30	9.23	0.27	< .05
Health insurance											
Private	28.92	0.45	77.57	0.42	47.28	0.49	15.89	0.37	7.61	0.27	< .001
Public	71.08	0.45	22.43	0.42	52.72	0.49	84.11	0.37	92.39	0.27	< .001
Patient characteristics											
Admission type											
Elective	32.13	0.47	36.84	0.48	43.97	0.50	28.52	0.45	25.33	0.44	< .05
Emergency	35.15	0.48	28.90	0.45	27.90	0.45	37.75	0.49	40.35	0.49	< .05
Other	32.72	0.49	34.25	0.47	28.13	0.45	33.73	0.47	34.32	0.47	
Diagnosis											
Bacterial and viral infection	10.58	0.31	10.09	0.30	8.75	0.28	11.79	0.32	11.03	0.31	< .05
Diseases of the blood	15.90	0.37	17.69	0.38	19.44	0.40	17.84	0.38	11.56	0.32	< .001
Endocrine and metabolic	17.47	0.38	15.53	0.36	21.10	0.41	18.37	0.39	14.69	0.35	< .001
Circulatory system	6.59	0.25	6.13	0.24	6.40	0.24	7.64	0.27	6.10	0.24	
Nervous system	16.82	0.37	17.95	0.38	20.81	0.41	17.00	0.38	13.78	0.34	< .001
Congenital and chromosomal	11.96	0.32	11.22	0.32	13.21	0.34	11.52	0.32	11.95	0.32	
Digestive system	19.43	0.40	15.70	0.36	16.87	0.37	20.22	0.40	21.92	0.41	< .001
Genitourinary system	6.06	0.24	6.04	0.24	4.75	0.21	6.75	0.25	6.38	0.24	
Musculoskeletal system	9.03	0.29	8.46	0.28	8.06	0.27	11.08	0.31	8.28	0.28	< .01
Respiratory system	17.85	0.38	17.00	0.38	18.07	0.39	18.59	0.39	17.62	0.38	
Trauma, injury, poisoning	9.56	0.29	9.92	0.30	6.23	0.24	10.82	0.31	10.54	0.31	< .001
Other diagnoses	35.26	0.48	39.78	0.49	31.79	0.47	34.75	0.48	35.66	0.48	< .01
Length of stay	3.67	5.35	3.44	5.02	3.27	4.99	3.93	5.60	3.84	5.47	< .01
Pain-level	4.48	3.51	4.12	3.49	4.01	3.42	4.74	3.58	4.68	3.47	< .01

Source: Data are from the 2013–2018 electronic medical records and the 2014–2018 American Community Survey (ACS)

Asterisks indicate significance difference evaluated using two-tailed independent means *t*-test

Results from our multilevel linear regression models predicting BMI *z*-scores are shown in Table 4. Model 1 partitions the total variation in BMI *z*-scores into within- and between-neighborhood variance. Model 2 only included the child/family-level characteristics (age, sex, race/ethnicity, insurance type, admission type, diagnosis, length of stay, patient-reported pain levels). Model 3 includes the LPA-generated neighborhoods. Fully specified Model 4 includes the interaction terms by race/ethnicity and neighborhood profiles. The random effects estimated across all models indicate significant variation in pediatric BMI *z*-scores across neighborhoods.

**Table 4:** Multilevel linear regression models predicting BMI *z*-scores; *N* = 8011 in 672 census tracts

	Model 1		Model 2		Model 3		Model 4	
	Coeff	95% CI	Coeff	95% CI	Coeff	95% CI	Coeff	95% CI
Intercept	0.36***	0.29–0.42	0.09	− 0.07–0.26	− 0.05	− 0.35–0.73	− 0.14	− 0.43–0.16
Patient-level								
Sociodemographic								
Age			0.03***	0.02–0.03	0.03***	− 0.14–0.02	0.03***	0.02–0.03
Sex (girl, ref)								
Boy			− 0.07	− 0.15–0.01	− 0.06	− 0.14–0.02	− 0.06	− 0.14–0.02
Race/ethnicity (Latinx, ref)								
Non-Latinx White			− 0.32***	− 0.44– − 0.19	− 0.30***	− 0.46– − 0.13	− 0.15	− 0.45–0.15
Non-Latinx Black			− 0.34**	− 0.58– − 0.09	− 0.39**	− 0.69– − 0.08	− 0.25	− 0.83–0.33
Asian			− 0.56***	− 0.72– − 0.39	− 0.48***	− 0.70– − 0.27	− 0.23	− 0.65–0.20
Other Race			− 0.10	− 0.22–0.02	− 0.07	− 0.23–0.10	0.11	− 0.22–0.45
Patient characteristics								
Health insurance (private provider, ref)								
Public provider			0.15**	0.05–0.26	0.10*	− 0.00–0.22	0.09	− 0.03–0.21
Admission type (elective, ref)								
Emergency			0.04	− 0.06–0.15	0.04	− 0.06–0.14	0.04	− 0.06–0.14
Other			− 0.10	− 0.15–0.07	− 0.05	− 0.15–0.06	− 0.05	− 0.15–0.06
Diagnosis (endocrine, nutritional. and metabolic diseases, ref.)								
Bacterial and viral infection			0.07	− 0.05–0.19	0.07	− 0.05–0.20	0.08	− 0.04–0.20
Diseases of the blood			− 0.12	− 0.24–0.01	− 0.11	− 0.23–0.01	− 0.11	− 0.23–0.01
Circulatory system			0.02	− 0.13–0.18	0.03	− 0.13–0.19	0.04	− 0.12–0.19
Nervous system			0.05	− 0.07–0.16	0.08	− 0.04–0.19	0.08	− 0.04–0.20
Congenital and chromosomal			− 0.33***	− 0.46– − 0.20	− 0.35***	− 0.48– − 0.21	− 0.35***	− 0.48–0.22
Digestive system			− 0.02	− 0.12–0.07	− 0.02	− 0.12–0.07	− 0.04	− 0.13–0.06
Genitourinary system			0.19*	0.04–0.35	0.16*	0.01–0.32	0.16*	0.01–0.32
Musculoskeletal system			− 0.15*	− 0.28– − 0.01	− 0.14*	− 0.28– − 0.01	− 0.15*	− 0.28– − 0.01
Respiratory system			− 0.09	− 0.20–0.02	− 0.09	− 0.19–0.02	− 0.09	− 0.19–0.02
Trauma, injury, poisoning			0.05	− 0.09–0.19	0.05	− 0.09–0.19	0.06	− 0.08–0.19
Other diagnoses			0.05	− 0.05–0.15	0.05	− 0.05–0.14	0.05	− 0.05–0.14
Length of stay			− 0.01***	− 0.02– − 0.01	− 0.01***	− 0.02– − 0.01	− 0.01**	− 0.02– − 0.01
Pain levels			0.04***	0.03–0.05	0.04***	0.02–0.05	0.03***	0.02–0.05
Neighborhood-level								
Neighborhood (Middle-Class, ref)								
Advantaged					− 0.21	− 0.42–0.01	0.01	− 0.43–0.45
Working-Class					0.06	− 0.12–0.23	0.12	− 0.12–0.35
Disadvantaged					0.21*	0.01–0.40	0.35**	0.11–0.60
Neighborhood average race/ethnicity					0.07	− 0.01–0.15	0.05	− 0.37–0.13
Neighborhood average health insurance					0.05	− 0.23–0.33	0.07	− 0.21–0.35
Interaction effects (Latinx*Middle-Class, ref.)								
Non-Latinx White*Advantaged							− 0.28	− 0.82–0.26
Non-Latinx White*Working-Class							− 0.20	− 0.62–0.23
Non-Latinx White*Disadvantaged							− 0.10	− 0.56–0.36
Non-Latinx Black*Advantaged							− 0.27	− 1.22–0.68
Non-Latinx Black*Working-Class							− 0.16	− 0.90–0.58
Non-Latinx Black*Disadvantaged							0.10	− 0.92–1.11
Asian*Advantaged							− 0.31	− 0.95–0.34
Asian*Working-Class							− 0.17	− 0.71–0.37
Asian*Disadvantaged							− 0.76*	− 1.43– − 0.08
Other Race*Advantaged							− 0.27	− 0.82–0.29
Other Race*Working-Class							0.04	− 0.39–0.46
Other Race*Disadvantaged							− 0.44*	− 0.86– − 0.01
Random effects								
Intercept	0.43***	0.36–0.51	0.26***	0.20–0.33	0.42***	0.34–0.51	0.41***	0.34–0.50
BMI *z*-scores	1.77***	1.71–0.82	1.79***	1.73–1.87	1.65***	1.58–1.72	1.64***	1.58–1.71

Source: Data are from the 2013–2018 electronic medical records and the 2014–2018 ACS

^***^p < 0.001, **p < 0.01, *p < 0.05

In Model 1, intraclass correlation from an unconditional model reveals that around 20% of the variation in children’s BMI *z*-scores is attributed between neighborhoods. In Model 2, we see that non-Latinx white (− 0.32; 95% CI: − 0.44 −  − 0.19), non-Latinx black (− 0.34; 95%CI: − 0.58 −  − 0.09), and Asian children (− 0.56; 95%CI: − 0.72 −  − 0.39), relative to Latinx, have lower BMI *z*-scores. Each additional year in age significantly increased BMI *z*-scores by 0.03 SD units (95% CI: 0.02 − 0.03). Publicly insured children (0.15; 95% CI: 0.05 − 0.26) and those with diseases of the genitourinary system (0.19; 95% CI: 0.04 − 0.35) and higher pain levels (0.04; 95% CI: 0.03 − 0.05) have higher BMI *z*-scores, whereas children with longer lengths of stay (− 0.01; 95% CI: − 0.02 −  − 0.01), congenital malformations, deformations and chromosomal abnormalities (− 0.33; 95% CI: − 0.46 −  − 0.20), and diseases of the musculoskeletal system and connective tissues (− 0.15; 95% CI: − 0.28 −  − 0.01) have lower BMI *z*-scores. In Model 3, accounting for neighborhood profiles slightly attenuates the racial/ethnic and socioeconomic differences in BMI *z*-scores indicating that some of the lower BMI *z*-scores for non-Latinx white, non-Latinx black, and Asian children, relative to Latinx, and higher BMI *z*-scores for publicly insured children, are due to the neighborhood environment. Model 3 largely mirrors Model 2 in other child/family-level factors and further shows that higher levels of neighborhood disadvantage independently associate with higher BMI *z*-scores. More specifically, children housed in Disadvantaged neighborhoods have 0.19 SDs higher BMI (95% CI: 0.02 − 0.36) compared to those living in Middle-Class communities. Finally, we examined whether race/ethnicity moderates the influence of neighborhood context on BMI *z*-scores. Model 4 indicates an interaction between neighborhoods and race/ethnicity. Specifically, we find that Latinx children living in Middle-Class neighborhoods have higher BMI *z*-scores than Asian (− 0.79; 95% CI: − 1.47 − 0.12) and Other Race (− 0.47; 95% CI: − 0.89 −  − 0.05) children living in the most disadvantaged communities.

## Discussion

This is the first study to date that employed LPA to detail how neighborhood context matters differently for children’s body weight status in a predominantly Latinx sample. We extracted EMR data from pediatric patients living in Southern California, a geographic setting with the largest Latinx population in the USA [42]. Importantly, we controlled for comorbid conditions like endocrine, nutritional, and metabolic diseases, as well as chromosomal, cardiovascular, pulmonary, neurological, musculoskeletal, and digestive system disorders that otherwise would bias estimates [7, 14–23]. Specifically, we generated neighborhood typologies based on social and economic indicators extracted from the ACS and found that the residential socioeconomic environment is associated with heightened BMI *z*-score. Our finding is consistent with published studies, but other researchers failed to account for underlying diagnoses that could introduce bias. Thus, in line with our expectations, using a diverse sample and accounting for important comorbid conditions, we show that a child’s neighborhood of residence independently associates with higher BMI *z*-scores. This could be driven by the overall higher incidence of overweight and obesity in the USA among Latinx youth, combined with compounding neighborhood-level determinants such as the absence of a health-promoting infrastructure that led to the greater prevalence of higher BMI [2–4, 31–37].

Our findings support previous reports that race/ethnicity moderates the association between LPA-constructed neighborhoods and children’s body weight. Specifically, here, we found that Latinx children living in more advantaged neighborhoods had higher BMI *z*-scores than Asian and Other children residing in the most disadvantaged communities. These results are troublesome given that a wealth of literature supports the notion that with affluence a child’s risk of presenting with obesity declines. Vis-à-vis individual-level indicators, the variation in children’s body weight associates with age, sex, race/ethnicity, SES, physical activity level, screen-time viewing, and sleep duration/bedtime [56–59]. Family-level, obesity-relevant factors include family physical activity, family mealtimes, food insecurity, and the income-to-need ratio [60–62]. The relationship among individual characteristics, in-home practices, and child obesity, however, is complicated by the influence of neighborhood-level descriptors [31–37]. For example, neighborhood median income modifies the association between a child’s BMI *z*-score and proximity to fast-food restaurants [63]. More specifically, the proximity to “unhealthy” food establishments has a stronger adverse effect on body weight status in lower-income neighborhoods, compared to the effect on residents of more affluent communities [63]. Thus, we need to optimize and implement a range of long-term, multicomponent intervention programs that focus on the early prevention of obesity. For example, a 3-year-long multilevel approach to reduce obesity among low-income, primarily Latinx children in Northern California, did show some promise for reducing weight gain [64]. Also, it is noteworthy that combined nutrition–physical activity initiatives, like *The California Endowment’s Healthy Eating, Active Communities* program, can be effective at preventing and reducing childhood obesity in low-income communities [65].

Published studies do offer insight into community-level factors that enhance susceptibility to obesity among children from socially vulnerable groups. For example, neighborhood safety influences the amount of physical activity and screen time [66, 67]. Also, poor adherence to physical activity recommendations among low-income Latinx women is a predictor of excessive gestational weight gain, which is independently associated with infant birthweight percentile [68, 69]. With regard to children, racial/ethnic differences exist across different groups in terms of physical activity and sedentary behaviors overall, and Latinx, compared to non-Latinx Black and non-Latinx White, children were found to participate in the least physical activity [70–73]. Crucially, the neighborhood context does impact girls and boys in a different way in terms of health benefits [31]. Relatedly, similar findings have been reported with regard to race/ethnicity. In Los Angeles County, for example, WIC-participating children across levels of neighborhood poverty are less likely to develop obesity, yet the prevalence of obesity remains higher among Latinx WIC-enrolled children compared to those who are non-Latinx White, non-Latinx Black, or Asian [74, 75]. Additionally, the neighborhood’s social and cultural fabric is yet another obesogenic factor. Indeed, the interplay of social group norms and networks like peers, school teachers, and digital mass media exposure informs acceptable ranges of body size and contributes to weight gain among Latinx youth [76–78]. Overall, data show that there is an immense disparity in the rates of pediatric overweight and obesity among racial/ethnic groups [4, 79]. Hence, intervention programs and public policies should include a multicomponent approach that targets parental behaviors, family patterns, household conditions, and the built and social infrastructure of communities [64, 65].

Our study, though informative, is not without limitations. We are constrained by the limited individual- and family-level variables available in the EMR data that are typically used for administrative purposes. For example, prior work indicates that children of immigrant parents present with higher levels of overweight, but we do not have information on immigration status nor origin group [80, 81]. Still, we included all covariates available to us in the EMR data known to associate with children’s body weight. Moreover, we use a public–private insurance dichotomization as a proxy measure for SES. We acknowledge that it is not ideal to use insurance type as a proxy for SES, but publicly provided health care coverage like Medicaid is only obtainable by children who meet stringent income criteria, with the exception of those with certain medical conditions [82]. Still, insurance coverage does have reasonable validity and reliability when used as a marker for individual-level SES [31, 32, 46–48]. Other scholars should test the reliability of our findings by using a more comprehensive set of individual- and family-level covariates known to associate with children’s body weight, including but not limited to nativity status, cultural differences, or parental education and income. Similarly, the cross-sectional nature of our data that are restricted to Los Angeles County and Orange County in Southern California limits the scope of our analyses. Nonetheless, this geographic setting has the largest Latinx population in the USA, which provides a unique opportunity to estimate the differential impacts of comprehensive neighborhood profiles on BMI *z*-scores in a diverse group of children [42]. Also, we used census tracts to represent neighborhoods. Census tracts are by no means a perfect operationalization of residential environments, but they remain a useful spatial entity available to us in the approximation of a neighborhood [30–32, 43]. Certainly, using addresses instead of zip codes provides a more robust spatial unit of analysis [37]. Finally, we examined the influence of distinct neighborhoods on children’s BMI *z*-scores. Although BMI is a simple measure, BMI categories are reasonably good for diagnosing pediatric obesity, especially when height and weight measures are collected objectively [83, 84]. Indeed, for our current study, height and weight were measured by trained healthcare workers.

Here, we sought to understand the pathways that underly the development of increased body weight in children by applying novel methods to a rich data source. Specifically, we used geocoded EMR data from pediatric patients to identify potential strategic targets for obesity prevention among Latinx youth residing in neighborhoods located in Los Angeles County and Orange County of Southern California. Our findings underscore the complex relationship between community racial/ethnic composition and body weight status during childhood. Indeed, here, we confirmed the significance of the place of residence on children’s body weight by focusing on the differential influence of race/ethnicity and neighborhood socioeconomic context. The results of our analyses raise critical questions for future research and represent opportunities for policy to help children maintain a healthy weight.

## Data Availability

Patient data are protected and are not available due to data privacy laws. Neighborhood data are available from the United States Census Bureau at https://www.census.gov/programs-surveys/acs/.
